# The Development of Accuracy and Fluency in Second Language (L2) Speaking Related to Self-Efficacy Through Online Scaffolding: A Latent Growth Curve Modeling Analysis

**DOI:** 10.1007/s10936-023-09950-7

**Published:** 2023-04-06

**Authors:** Cha Li, Lawrence Jun Zhang

**Affiliations:** 1 School of Foreign Languages, Northeast Normal University, Changchun, China; 2 Faculty of Education and Social Work, The University of Auckland, Auckland, New Zealand

**Keywords:** Accuracy, Fluency, Self-efficacy, L2 speaking, LGCM

## Abstract

This longitudinal study made the first attempt to employ Latent Growth Curve Modeling to analyze the development of L2 speaking accuracy and fluency through online scaffolding as well as the dynamic relationship between L2 speaking performance and self-efficacy. From the perspective of Complex Dynamic Systems Theory, it tracked the development of 45 Chinese undergraduates’ English-speaking accuracy, fluency, self-efficacy for accuracy (SEA) and self-efficacy for fluency (SEF) over one semester of online teaching (six observations). Results show that speaking accuracy, SEA and SEF all improved significantly, but speaking fluency did not; these four variables all developed in non-linear trajectories, and the greatest growth of accuracy, SEA and SEF all took place at Time 2; there existed significant individual differences in the initial levels of fluency, SEA and SEF, and in the change rates of SEA; a higher initial level of accuracy was related to a greater increase in SEA and a greater decrease in growth rates with time. These findings provide evidence for non-linearity, variability and inter-individual differences in the development of L2 speaking and self-efficacy through online scaffolding, and partly confirm the dynamic relations between self-efficacy and L2 performance. Pedagogical implications for online scaffolding are also discussed.

## Introduction

Second language (L2) speaking development, a valuable but long-neglected topic (Lowie & Verspoor, 2022), has gained research attention in the recent decade (Ferrari, 2012; Polat & Kim, 2014; Sun & Zhang, 2022; Vercellotti, 2017; Yu & Lowie, 2020). While these studies confirmed the view of Complex Dynamic Systems Theory (CDST) that intra-individual variabilities and inter-individual differences exist in complexity, accuracy and fluency (CAF) development, the dynamic relationship between L2 speaking CAF development and individual difference factors was rarely investigated.

L2 self-efficacy, as one important individual difference factor, was proven by many cross-sectional studies to positively correlate with and predict L2 performance (e.g., Hsieh & Schallert, 2008; Mahyuddin et al., 2006; Mills et al., 2007; Noorollahi, 2021; Truong & Wang, 2019). Self-efficacy refers to one’s belief about whether or not one can perform a task successfully (Bandura, 1977, 1997). L2 self-efficacy itself is not static and has been proven to change with time, as documented in some longitudinal studies (Fryer et al., 2022; Kyo, 2022; Leeming, 2017; Shirvan et al., 2018; Zhang et al., 2020); so it is necessary that L2 self-efficacy be investigated longitudinally so that the dynamic relationship between L2 development and self-efficacy can be examined. Unfortunately, no previous studies have ever analyzed whether the development of accuracy, fluency and complexity in L2 speaking is in relation to the development of self-efficacy.

Due to the influence of the COVID-19 pandemic, many universities have been offering otherwise traditional courses online, in which teachers provide necessary support to students as scaffolding via online teaching software. As is evident, such a mode of delivery is rather different and does not afford any opportunity for face-to-face interaction. More significantly, longitudinal studies on the development of accuracy, fluency and complexity in L2 speaking through online scaffolding are non-existent in the literature.

The current study intends to fill these gaps by tracking and analyzing 45 Chinese undergraduates’ oral data and self-efficacy scores over one semester of online teaching. The three dimensions of accuracy, fluency and complexity were sometimes fully reported (Ferrari, 2012; Vercellotti, 2017), and sometimes partially focused (Polat & Kim, 2014; Sun & Zhang, 2022; Yu & Lowie, 2020). Due to limited space, we only focus on accuracy and fluency. Accuracy refers to the extent to which L2 production conforms to native speakers’ use of grammar and vocabulary, and fluency means the extent to which L2 production is similar to that of native speakers in temporal and hesitating features (Ellis, 2003, 2008; Ellis & Barkhuizen, 2005; Lennon, 1990; Skehan, 1996; Wolfe-Quintero et al., 1998). Both accuracy and fluency are effective indicators of second language performance and development (Larsen-Freeman, 2009; Skehan, 2009). This study aims to explore the dynamic relationship between self-efficacy and L2 speaking accuracy and fluency development.

## Literature Review

### CDST and Latent Growth Curve Modeling (LGCM)

From the CDST perspective, non-linearity and inter-individual differences in L2 development and individual learner factors should be put in the center, and the relationship between language development and learner factors should be viewed in a dynamic way (Larsen-Freeman, 1997, 2006, 2009, 2020; Larsen-Freeman & Cameron, 2008; Lowie & Verspoor, 2019). The current study took this perspective and thus attached great importance to the following three aspects.

Firstly, non-linearity is one important feature of complex systems, which can only be studied through process-oriented research based on multiple observations (Lowie, 2017; van Geert & Steenbeek, 2005). Accuracy, fluency and complexity variables are developmental in nature (Larsen-Freeman, 2009; Norris & Ortega, 2009), and L2 self-efficacy was found to be dynamic (Fryer et al., 2022; Kyo, 2022; Leeming, 2017; Shirvan et al., 2018; Zhang et al., 2020), so they need to be tracked longitudinally. However, traditional modeling usually limits non-linearity to preset polynomial functions, which may not fit the complex and dynamic development. The current study intends to address this issue by using free time scores Latent Growth Curve Modeling (LGCM).

Secondly, inter-individual differences are considered as information, instead of noise from the perspective of CDST (Larsen-Freeman & Cameron, 2008) and similar learners may have very different trajectories of learning (Lowie & Verspoor, 2019). Some previous studies have found inter-individual differences in accuracy, fluency and complexity (Larsen-Freeman, 2006; Yu & Lowie, 2020) and self-efficacy development (Leeming, 2017; Shirvan et al., 2018). This study uses LGCM to analyze whether there are inter-individual differences in the initial levels and change rates of L2 speaking and self-efficacy.

Thirdly, in complex dynamic systems, the relationship between factors and system development is dynamic (Larsen-Freeman, 1997, 2006, 2009, 2020; Larsen-Freeman & Cameron, 2008; Lowie & Verspoor, 2019), which cannot be revealed by cross-sectional studies. One cross-sectional study proved that L2 speaking self-efficacy correlated with and predicted speaking performance (Asakereh & Dehghannezhad, 2015), but a longitudinal study of speaking performance and self-efficacy did not confirm the influence of self-efficacy on speaking performance (Zhang et al., 2020). So far, it seems that no research has ever explored whether the change rates of accuracy, fluency and complexity in L2 speaking and self-efficacy relate with each other during the process of development, which responds to CDST’s emphasis on dynamic relations. This study uses LGCM with parallel processes to explore the relationship between L2 speaking and self-efficacy.

LGCM is a suitable method to analyze longitudinal data from a CDST perspective (Hiver & Al-Hoorie, 2019). It makes it possible to model not only the trajectory of the whole group, but also of individuals, coping with the issue of inter-individual differences (Hiver & Al-Hoorie, 2019). Besides traditional linear and non-linear growth model based on polynomial functions, LGCM supports free time scores, in which time scores are set free and estimated based on the real data, instead of relying on preset polynomial functions and trajectories (Wang, 2004). Free time scores can help judge whether the trajectory is linear or not, and also help calculate variations between observations (Wang, 2009). Besides, LGCM with parallel growth processes can help explore the relationship between two or more outcome variables in the process of development (Muthén & Muthén, 1998–2017).

Some recently published studies have used LGCM to analyze the development of language learner factors such as emotions, grit and self-efficacy (Fryer et al., 2022; Kyo, 2022; Shirvan et al., 2018, 2021), but no previous study has ever used LGCM to analyze accuracy, fluency and complexity development in L2 speaking, or the relationship between oral accuracy, fluency and complexity and self-efficacy. To fill this gap, the current study made the first attempt to use LGCM to analyze the development of English speaking accuracy and fluency and the dynamic relationship between English speaking self-efficacy and oral English development.

### Development of Accuracy and Fluency in L2 Speaking

Existing studies of accuracy and fluency development in L2 speaking mainly focused on two different aspects: group development (Tonkyn, 2012; Vercellotti, 2017; Yu & Lowie, 2020) and individual trajectories (Evans & Larsen-Freeman, 2020; Ferrari, 2012; Polat & Kim, 2014; Yu & Lowie, 2020). Previous analysis of group data have shown improvement in accuracy or fluency (Tonkyn, 2012; Vercellotti, 2017; Yu & Lowie, 2020). For example, Tonkyn (2012) compared 24 postgraduates’ accuracy, fluency and complexity in English speech before and after a 10-week course in the UK and found improvement in both accuracy and fluency measures. Vercellotti (2017) employed Hierarchical Linear and Non-linear Modeling (HLM) to analyze 66 English as a second language (ESL) learners’ oral data over 3–10 months and found improvement in accuracy and fluency. Yu and Lowie (2020) compared a group of 10 Chinese undergraduates’ oral performance over 12 weeks and found significant improvement in accuracy as well. These studies show that accuracy is likely to grow in one semester for learners who took courses in target-language context (Tonkyn, 2012) and those who took courses in their own country (Yu & Lowie, 2020), but whether fluency could advance in one semester for learners studying English in their own country remains unknown.

Longitudinal case studies based on individuals’ intense data usually supported non-linear development (Evans & Larsen-Freeman, 2020; Ferrari, 2012; Polat & Kim, 2014; Yu & Lowie, 2020) and inter-individual differences (Ferrari, 2012; Yu & Lowie, 2020). Ferrari (2012) observed four L2 learners’ Italian speech over three years and found improvement in accuracy and fluency in the long run, with some fluctuations during the process and inter-individual differences in rates. Polat and Kim (2014) tracked the English speech of an untutored Turkish immigrant over one year and found dynamic variations during the process but no general improvement in accuracy. Besides group data analysis, Yu and Lowie (2020) also selected two learners’ data to observe individual development and found high degree of intra-individual variability and inter-individual differences in accuracy development. Evans and Larsen-Freeman (2020) tracked an untutored learner’s English syntax development over 30 weeks and confirmed the non-linear nature of accuracy and fluency development.

Based on previous group data analysis (Tonkyn, 2012; Yu & Lowie, 2020), accuracy is very likely to experience advancement for EFL learners in one semester, and based on case studies (Evans & Larsen-Freeman, 2020; Ferrari, 2012; Polat & Kim, 2014; Yu & Lowie, 2020), the development of accuracy and fluency should be non-linear. However, previous studies on L2 speaking accuracy and fluency development were mainly based on offline courses (Ferrari, 2012; Tonkyn, 2012; Vercellotti, 2017; Yu & Lowie, 2020), whether accuracy and fluency could improve through online scaffolding and how they develop in the context of online teaching has not been investigated yet. Besides, previous studies based on group data (Tonkyn, 2012; Vercellotti, 2017; Yu & Lowie, 2020) were not able to present the “real” non-linearity due to the limitation of analysis methods. Traditional non-linear models based on polynomial functions are limited in describing real trajectories (Meredith & Tisak, 1990). Free time scores LGCM used in the current study can solve this problem by setting free time scores without the restriction of preset trajectories (Wang, 2004).

### Development of L2 Self-Efficacy

In the field of SLA, studies of L2 self-efficacy development are either “result-oriented” (Mills, 2009) or “process-oriented” (Fryer et al., 2022; Kyo, 2022; Leeming, 2017; Piniel & Csizér, 2015; Shirvan et al., 2018).

Earlier studies are more result-oriented because the initial motive for tracking self-efficacy was to verify the effects of some specific teaching methods. For instance, Mills (2009) compared 46 college students’ L2 French self-efficacy before and after a semester’s project-based course and found significant improvement.

Recent years have witnessed more process-oriented studies (Fryer et al., 2022; Kyo, 2022; Leeming, 2017; Piniel & Csizér, 2015; Shirvan et al., 2018). Despite a small number, these studies covered both general L2 self-efficacy and self-efficacy for specific writing or speaking skills. As for general L2 self-efficacy, Shirvan et al. (2018) investigated 367 undergraduates’ English self-efficacy 4 times over a semester and by using linear LGCM found significant increases with inter-individual differences. Two recently published studies (Fryer et al., 2022; Kyo, 2022) also used linear LGCM to analyze the development of L2 self-efficacy. Fryer et al. (2022) found a decrease in 1184 Japanese undergraduates’ self-efficacy for English course (3 times) during one semester; and Kyo (2022) found a slight increase in 4501 South Korean secondary school students’ English self-efficacy over three years. These two studies used LGCM results as a basis for their further analysis and did not focus on non-linearity or inter-individual differences in L2 self-efficacy development. As for L2 writing self-efficacy, Piniel and Csizér (2015) tracked 21 English majors’ writing self-efficacy over a semester (6 times) and the results from the questionnaire showed that self-efficacy decreased, but their retrospective interview data showed an increase. It is noteworthy that Piniel and Csizér (2015) adopted free time scores LGCM to analyze the development of several factors, but the model did not fit well the data of self-efficacy and was not reported. As for L2 speaking self-efficacy, Leeming (2017) analyzed 77 Japanese students’ English speaking self-efficacy over an academic year. Hierarchical Linear Modeling showed a growth in self-efficacy with individual differences in rates of growth. Despite the use of advanced techniques in analyzing longitudinal data in these studies (Fryer et al., 2022; Kyo, 2022; Leeming, 2017; Piniel & Csizér, 2015; Shirvan et al., 2018), the non-linear feature of L2 self-efficacy development, especially L2 speaking self-efficacy development has yet to be fully explored.

Despite different conclusions, some studies indicated the possible improvement of L2 self-efficacy over a semester (Mills, 2009; Shirvan et al., 2018) and the possible improvement of L2 speaking self-efficacy over an academic year (Leeming, 2017), but whether L2 speaking self-efficacy could increase through one semester of online scaffolding is unclear. Furthermore, it seems that no previous studies have focused on self-efficacy for English speaking accuracy and fluency specifically.

### L2 Self-Efficacy and L2 Performance

L2 self-efficacy was proven to correlate with and predict the overall L2 achievement in many studies based on cross-sectional data (e.g., Hsieh & Schallert, 2008; Mahyuddin et al., 2006; Mills et al., 2007; Noorollahi, 2021; Truong & Wang, 2019). There are also studies focusing on one aspect of language skills. For instance, L2 reading self-efficacy was found to predict reading achievement (Ghonsooly & Elahi, 2010); L2 writing self-efficacy could predict writing performance (Woodrow, 2011); and L2 speaking self-efficacy was also proven to correlate with and predict speaking performance (Asakereh & Dehghannezhad, 2015). All the above studies established a strong relationship between L2 self-efficacy and L2 achievement or performance. However, previous studies usually used rated scores to measure achievement or performance (Asakereh & Dehghannezhad, 2015; Noorollahi, 2021; Truong & Wang, 2019), and no study seems to have explored the relation between accuracy and fluency variables and relevant self-efficacy.

Longitudinal studies on the dynamic relationship between L2 self-efficacy and L2 performance are very limited. Zhang et al. (2020) explored the relationship between 82 Chinese undergraduates’ self-efficacy and public speaking performance in 4 tasks over one semester. ANOVA showed significant improvement in both self-efficacy and speaking performance, but path analysis showed that self-efficacy did not predict performance in a significant way. This study tracked the development of self-efficacy and public speaking performance longitudinally, but did not analyze whether the initial levels and change rates of self-efficacy and speaking performance related over the observation period. One recent longitudinal study found that the initial level and change rates of L2 self-efficacy could predict L2 achievements (Kyo, 2022), but it did not track L2 performance dynamically, so whether their change rates relate with each other was not known.

The cross-sectional study (Asakereh & Dehghannezhad, 2015) and the longitudinal study (Zhang et al., 2020) drew conflicting conclusions about the relationship between L2 speaking performance and L2 speaking self-efficacy, and no previous studies seem to analyze whether the development of L2 speaking self-efficacy relates with the development of L2 speaking, especially in specific dimensions of accuracy and fluency.

As reviewed above, previous studies did not analyze the development of L2 speaking accuracy and fluency by using LGCM, using which can better address the problem of non-linearity and inter-individual differences. More importantly, no study has ever focused on the development of self-efficacy for speaking accuracy and fluency specifically and no research has been conducted to examine the dynamic relationship between L2 speaking CAF development and self-efficacy development nor the development of L2 speaking accuracy, fluency and self-efficacy through online scaffolding. Therefore, we have made the first attempt to use LGCM to analyze 45 Chinese undergraduates’ longitudinal oral data and self-efficacy scores over one semester of online teaching (6 times) and aim to answer the following questions.

RQ1. How did students’ English speaking accuracy and fluency develop through online scaffolding over one semester? Were trajectories linear or non-linear? Were there inter-individual differences?

RQ2. How did students’ self-efficacy for accuracy (SEA) and self-efficacy for fluency (SEF) develop through online scaffolding over one semester? Were trajectories linear or non-linear? Were there inter-individual differences?

RQ3. Did the initial levels and change rates of accuracy and SEA relate during the observation period? Did the initial levels and change rates of fluency and SEF relate during the observation period?

## Materials and methods

### Participants

Based on the convenience sampling method, a total of 52 grade one Chinese undergraduates from two parallel English classes taught by the first author participated in this longitudinal study. After data cleaning, the full data of 45 students were finally included in the analysis. They were from various departments of the university (Table 1). They had learned English for 10 years before college and studied College English for one semester before this project. They had never visited any English-speaking country before, nor during the time when the research project was in progress. Most participants did not attach importance to oral English before college because the College Entrance Examination did not include speaking scores. According to a placement test at the beginning of the first semester, their English level was medium among all first-year students in the university.

**Table 1:** The demographic information of participants

Department	Male	Female	Total
	Number (Proportion)	Number (Proportion)	Number (Proportion)
Physics	1 (2.22%)	7 (15.56%)	8 (17.78%)
Chinese literature	1 (2.22%)	6 (13.33%)	7 (15.56%)
Biology	2 (4.44%)	4 (8.89%)	6 (13.33%)
Chemistry	1 (2.22%)	4 (8.89%)	5 (11.11%)
Geography	0	4 (8.89%)	4 (8.89%)
Mathematics	2 (4.44%)	2 (4.44%)	4 (8.89%)
Environment	1 (2.22%)	2 (4.44%)	3 (6.67%)
History	0	3 (6.67%)	3 (6.67%)
Education	0	2 (4.44%)	2 (4.44%)
Marxism	0	2 (4.44%)	2 (4.44%)
Psychology	1 (2.22%)	0	1 (2.22%)
Total	9 (20%)	36 (80%)	45 (100%)

### Online Scaffolding in the Present Study

In this university, College English, a common English proficiency course, was offered twice a week (180 min). Due to the impact of COVID-19, the university turned all courses into online courses in the second semester (the spring semester in 2020), when this study was conducted. DingTalk, a popular online teaching platform in China, was applied to deliver online lectures, assign tasks and interact with students through questions and answers; QQ, a popular social networking platform, which enables the teacher and students to send text, voice, pictures and document files in the class QQ group, was used to present orally or submit written work and provide teacher or peer feedback; online courses powered by UMOOCs (https://moocs.unipus.cn) were also utilized to provide scaffolding. Due to the limitation of the online teaching platform, group activities were replaced by new forms of activities in the QQ group. Since this is not an intervention study, ways of online scaffolding were just briefly presented here to give readers a better understanding of the research context. The typical procedure of a teaching unit is composed of three steps (3 times, 270 min), and ways of online scaffolding are described below with an example:

Step 1: Theme-based activity.

(Example: Book Recommendation)

Pre-activity scaffolding:

Content: the outline of a recommendation card.

Medium: DingTalk.

While-activity scaffolding:

Content: the template of oral book recommendation by using the card.

Medium: DingTalk.

Post-activity scaffolding:

Content: feedback on cards and oral recommendation.

Medium: QQ group.

Step 2: Text study.

Pre-reading scaffolding:

Content: Lead-in and cultural background.

Medium: UMOOCs (teaching video) and DingTalk (interaction).

While-reading scaffolding:

Content: Reading strategies, language points, genre, critical thinking.

Medium: UMOOCs (teaching video) and DingTalk (interaction).

Post-reading scaffolding:

Content: ways to draw mind maps of text structure.

Medium: DingTalk (for teaching) and QQ group (for presentation and feedback).

Step 3: Theme-based and text-related writing.

Pre-writing scaffolding:

Content: the way to write a book review and some samples.

Medium: DingTalk.

Post-writing scaffolding:

Content: feedback on students’ writing.

Medium: QQ group.

Different from classroom teaching, the online teaching could not monitor the learning process very effectively and thus attached more importance to feedback. In traditional classroom teaching, it is difficult for every student to give oral presentations for teacher and peer feedback, but the online scaffolding in this study enabled more adequate and individualized feedback from teachers and peers. It should be noted that this was not a specific speaking course, and no special training in accuracy or fluency was given. Compared with traditional or usual classroom teaching, the spontaneous oral production and natural communication was much less frequent, but the prepared oral production was more frequent and given more teacher and peer feedback, because every student could share his/her voice recording to the QQ group, where the voice stayed and could be heard repeatedly.

### Scale of Self-Efficacy for English Speaking Accuracy and Fluency

The scale was adapted from ESSS-CCS (Li & Sui, 2022), a scale designed by the first author to measure Chinese college students’ self-efficacy for English speaking. The original scale comprises five dimensions, including SEA (self-efficacy for accuracy) and SEF (self-efficacy for fluency). The scale was designed in accordance with the guide for constructing self-efficacy scale (Bandura, 2006), asking participants to rate their confidence about English speaking. All the statements are on an 11-point Likert scale because it was believed to predict performance better than a 5-point scale (Bandura, 2006; Pajares et al., 2001). The structural validity of the ESSS-CCS was examined in a sample of 800 Chinese college students (300 for exploratory factor analysis or EFA, and 500 for confirmatory factor analysis or CFA) and the reliability was high (Li & Sui, 2022).

The original data of SEA and SEF were again subjected to CFA in Mplus (Estimator = MLR). The model showed good fit to the data [X^2^ (df) = 10.481 (8), *p* = .233, CFI = 0.997, TLI = 0.995, RMSEA (90% CI) = 0.025 (0.000 − 0.061), PCLOSE = 0.849, SRMR = 0.018]. The estimated correlation between the two factors is 0.778. Table 2 presents the six items and CFA factor loadings (the scale was developed in Chinese, and the statements in the table are the translated version for readers’ convenience). CFA results show that these two dimensions can be taken independently from the original full scale and are valid in measuring self-efficacy for accuracy and fluency.

The reliability was tested using the whole sample of 800 questionnaires. It was high for both SEA (Cronbach α = 0.907) and SEF (Cronbach α = 0.878), and the overall reliability was also high (Cronbach α = 0.910). The reliability was also high when the scale was used in this longitudinal study (Table 3).

**Table 2:** Scale of Self-efficacy for English speaking accuracy and fluency CFA factor loadings.^a^

Factors	Items	Factor loadings	*p*	Std. error
SEA	1. When I speak English, I can use correct grammar (e.g. past tense, the third person singular, etc.)	0.832	< 0.001	0.021
	2. When I speak English, I can use correct sentence structure and word order.	0.914	< 0.001	0.015
	3. When I speak English, I can use correct words.	0.900	< 0.001	0.017
SEF	4. When I speak English, I can avoid silent pauses.	0.832	< 0.001	0.024
	5. When I speak English, I can avoid unnecessary repetitions.	0.865	< 0.001	0.025
	6. When I speak English, I can avoid self-repair.	0.866	< 0.001	0.026

^a^ SEA = self-efficacy for accuracy; SEF = self-efficacy for fluency. n = 500

**Table 3:** Reliability (Cronbach α) of the scale used in this study.^b^

Scale (items)	Time 1	Time 2	Time 3	Time 4	Time 5	Time 6
SEA (3)	0.874	0.850	0.919	0.911	0.923	0.852
SEF (3)	0.808	0.858	0.943	0.942	0.905	0.908
Overall scale (6)	0.866	0.872	0.914	0.936	0.931	0.904

^b^ SEA = self-efficacy for accuracy; SEF = self-efficacy for fluency. n = 45

### Data Collection

In order to track the development of students’ accuracy, fluency and self-efficacy, the first author collected data every 3 weeks during the break time of classes by utilizing online teaching facilities. All participants were well informed of and agreed with the process of data collection. They understood that all data collection was for research purposes and not related to their grades. Data were collected on Mondays of Weeks 1, 4, 7, 10, 13 and 16.

Students were asked to prepare two devices: one computer for the DingTalk camera and one smartphone for recording oral data and uploading the audio file to QQ. They were asked to enter the virtual classroom in DingTalk, turn on their cameras with silent mode, and adjust to a proper position so that the researcher and two teaching assistants could see their behaviors clearly during preparation and speaking. To familiarize students with the process, pilot data collection was conducted before the first week, in which all tasks were fake and meaningless in content (to avoid any influence on the formal data collection). For instance, the oral task was replaced by “making any sound”. The formal procedure for data collection was as follows:

First, students were asked to use smart phones to fill in the online SEA and SEF scales (the order of items were randomized every time) powered by www.wjx.cn, a website similar to SurveyMonkeys that is used for conducting online surveys. After all of them completed the survey and typed “1” in DingTalk to show they were ready, they were assigned an oral task (in typed words) through DingTalk. They had one minute to prepare without taking notes or referring to dictionaries. After one minute, the researcher asked them to begin their talk. They were told to talk for about two minutes and record their speech with their smartphones. Finally, they were asked to name their audio files with their codes and instantly send the files to the researcher’s QQ account. The whole process was monitored carefully by the researcher and two teaching assistants (each monitored 9 students on one screen, 26 students per class). If any student did not follow the rule of preparation, his oral data were excluded. Since 7 students occasionally disobeyed the rule of preparation when taking oral tasks, finally 45 students’ full data were used in the current study.

This study is not an intervention study, and the main aim is to observe the natural development of accuracy, fluency and self-efficacy in the context of online teaching. Therefore, no specific training about accuracy or fluency was provided, but feedback on participants’ pronunciation was given back for their benefit.

The six oral tasks were chosen from the IELTS speaking test. They are monologic tasks of the same difficulty in terms of cognitive demands. Topic-given monologic tasks were often used in longitudinal studies (Vercellotti, 2017; Yu & Lowie, 2020). These students used to take an oral test (reading and topic-given monologic tasks) at the end of the first semester, so they were familiar with monologic tasks used in the current study. All the topics were rated by other 60 first-grade undergraduates in the same university, and the difficulty level of the six topics was similar, ranging from 2.483 to 2.550 (1 = very easy, 2 = easy, 3 = medium, 4 = difficult, 5 = very difficult). To minimize the topic effects, the order of topics in the first class was 1-2-3-4-5-6; while the order in the second class was 6-5-4-3-2-1. All the topics are listed below:

1. Describe a project or some work that you did with others.
2. Describe a happy event from your childhood that you remember well.
3. Describe a friend you have known for a long time.
4. Describe a situation when you needed some advice.
5. Describe a useful practical skill that you learned.
6. Describe an enjoyable family event that you attended.

For every topic, there were similar prompts, asking participants to talk about when, where, who, how and why. For example, the prompts for the first topic were: “You should say what the project or work was; when you did this; who was with you; how easy or difficult it was; and explain why you did this with others”.

### Transcription

All the 270 oral speech samples were transcribed verbatim in CHAT formatted for analysis using CLAN (MacWhinney, 2000). Utterances were divided according to As-unit principles, which are more suitable for oral data (Foster et al., 2000). AS-unit refers to an utterance “consisting of an independent clause, or sub-clause unit, together with any subordinate clauses associated with either” (Foster et al., 2000, p.365). Repetition was coded with [/] and self-revision was coded with [//] to help exclude ineffective words in later analysis. The transcription was done by the first author and checked by a research assistant (a Ph.D. student of applied linguistics). All disagreements in transcription were resolved after discussion. Then we randomly chose 30 transcripts (more than 10% of all the samples) and coded inaccurate As-units. The inter-coder agreement was high (94.6%). Then the author and the assistant each coded 120 transcripts. Finally, we checked each other’s coding and reached an agreement after referring to spoken corpora (COCA and BNC).

### Measures for Accuracy and Fluency

A widely used measure “percentage of error-free As-units” (Vercellotti, 2012; Yu & Lowie, 2020) was applied to measure accuracy. This measure has a limitation of ignoring the number of errors in each As-unit, but we adopted it because coding error-free As-units causes less inconsistency than coding every error. Here error means “a linguistic form or combination of forms which, in the same context and under similar conditions of production, would, in all likelihood, not be produced by the speakers’ native speaker counterparts” (Lennon, 1991, p. 182). Both grammatical and lexical errors were considered, and incorrect As-units were judged by the first author and a research assistant based on English grammatical rules and spoken corpora (COCA and BNC). When there was self-repair, only the final version was considered.

The fluency measure is “number of syllables per minute” (Ellis & Barkhuizen, 2005). In this measure, only meaningful syllables were counted and disfluencies (repetitions and self-repairs) were excluded (Ellis & Barkhuizen, 2005). The pruned speech rate was proven to be a reliable global measure of fluency in some studies (Awwad & Tavakoli, 2022; Dabaghi Varnosfaderani et al., 2022). After transcription, disfluent words were excluded using CLAN, and the syllables were counted with the help of an online tool called Syllable Counter (www.syllablecount.com/#MOVEHERE), which counts syllables based on the U.S. English syllable count dictionary and lists words unidentified. Controversial and unidentified words were checked by the first author manually based on the recordings.

### Data Analysis

To answer the first two questions about the development of accuracy, fluency, SEA and SEF, LGCM (in Mplus) was used to analyze the longitudinal data of these four variables separately. Data were used for computing linear LGCM, non-linear LGCM (quadratic function) and free time scores LGCM.

In LGCM, the paths from intercept factors to observed variables are constrained to 1, and the paths from slope factors to observed variables indicate time scores (constrained to 0, 1, 2, 3… in linear LGCM with equal time intervals). In non-linear LGCM (quadratic function), a second-order slope factor is added. In free time scores LGCM, for the purpose of model recognition, at least two time scores should be set, one as 0, and another usually as 1 (Wang, 2004, 2009).

These models were compared and evaluated based on the following model fit indices: AIC BIC and SABIC (the smaller, the better); chi square and *p* value (*p* > .05 indicates good model fit); CFI and TLI (> 0.900 acceptable fit; >0.950 excellent fit) (Hu & Bentler, 1999). The best model was chosen based on a combination of these indices, and model results were reported. RMSEA and SRMR were not used, because according to a study (Taasoobshirazi & Wang, 2016) examining the performance of CFI, TLI, RMSEA and SRMR in latent growth models across various sample sizes and degrees of freedom, the rejection rates of correctly specified models in the case of small sample size (50 samples with 10–20 degrees of freedom) are very high when using RMSEA and SRMR. Therefore, RMSEA and SRMR are not recommended to be reported in models with such a small sample size (Taasoobshirazi & Wang, 2016).

The model results were reported in the following steps. Firstly, means of slope were used to judge the direction of change and whether the increase or decrease was significant. In free time scores LGCM, time scores were used to judge whether the development was linear or non-linear, and means of slope and time scores were combined to calculate variations between observations (Wang, 2004, 2009). For example, the variation of accuracy between Time 3 and Time 2 was 0.159 *(1.358-1.000) = 0.057. (0.159 was the mean of slope, 1.358 was the time score of accuracy at Time 3, and 1.000 was the time score of accuracy at Time 2). Finally, variances of intercept and slope were used to judge whether there were significant inter-individual differences in initial levels and rates of change.

To answer the third question about the relationship, LGCM with parallel processes of accuracy and SEA, and LGCM with parallel processes of fluency and SEF were built respectively. The covariance between two variables’ intercepts and slopes was used to determine whether the initial levels and change rates of these two variables related with each other. Since the model of fluency and SEF got a warning, SEF at Time 1 was put into free time scores LGCM of fluency as a covariate to find out whether the initial level of SEF influenced the initial level and change rates of fluency.

## Results

### Development of Accuracy and Fluency Through Online Scaffolding

The longitudinal data of accuracy and fluency were used to build linear, non-linear and free time scores LGCM. The model fit information is presented in Table 4 (linear and non-linear models of accuracy were warned “PSI is not positive definite” and non-linear LGCM of fluency was not identified). For accuracy, free time scores LGCM fit the data very well; for fluency, both linear and free time scores models fit well, but the latter fit better based on a combined consideration of these indices and the theoretical foundation. Therefore, the model results of free time scores LGCM of accuracy and free time scores LGCM of fluency are reported.

The mean initial level of the accuracy variable (percentage of error-free As-units) was 0.330, which was very low. There was a significant increase in accuracy (mean of slope = 0.159, *p* < .001) over the semester. Time scores were 0.000, 1.000, 1.358, 1.130, 1.531 and 1.049. If the development is linear, time scores should be 0, 1, 2, 3, 4, 5, because the time interval was equal. It is evident that accuracy developed in non-linear trajectories.

**Table 4:** The model fit information of LGCM of accuracy and fluency

Model	AIC		BIC	SABIC	X^2^ (df)	*p*	CFI	TLI
Free time scores LGCM of accuracy		-101.274	-74.174	-121.193	8.591 (12)	0.737	1.000	1.000
Linear LGCM of fluency		2354.638	2374.511	2340.031	19.489 (16)	0.244	0.989	0.990
Free time scores LGCM of fluency		2356.005	2383.105	2336.087	13.139 (12)	0.359	0.996	0.995

**Fig. 1:**
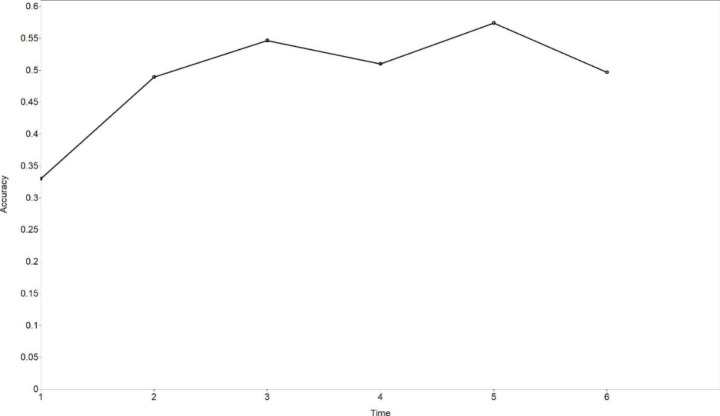
The estimated trajectory of accuracy (free time scores LGCM)

Variations between observations were calculated and presented in Table 5, showing that the greatest increase happened at Time 2 for accuracy, and the accuracy score fluctuated with smaller variations from Time 2 to Time 6. The estimated trajectory is shown in Fig. 1. Significant inter-individual differences were not found in the initial level (mean of variance = 0.017, *p* = .272) or change rates (mean of variance = 0.006, *p* = .539) of accuracy.

**Table 5:** Variations of accuracy, fluency and SEF between observations.^c^

	Time2-1	Time3-2	Time4-3	Time5-4	Time6-5
Accuracy	0.159	0.057	− 0.036	0.064	− 0.077
Fluency	− 0.174	7.136	0.522	4.122	-1.741
SEF	0.750	0.419	0.692	0.070	0.082

^c^ SEF = self-efficacy for fluency.

**Fig. 2:**
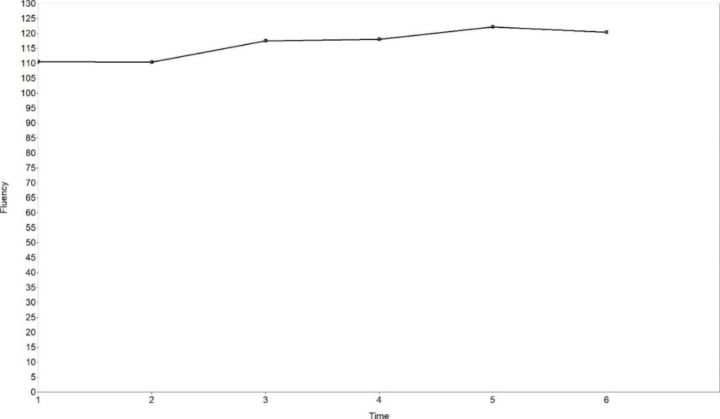
The estimated trajectory of fluency (free time scores LGCM)

The mean initial level of the fluency variable (number of syllables per minute) was 110.592, and the increase of fluency was not significant (mean of slope = 6.962, *p* = .155). Time scores were 0.000, − 0.025, 1.000, 1.075, 1.667 and 1.417, indicating non-linear development of fluency. Variations between observations were calculated based on means of slope and time scores (Table 5). The most evident increase of fluency happened at Time 3. The estimated trajectory is shown in Fig. 2. Significant inter-individual differences were found in the initial level of fluency (variance of intercept = 490.418, *p* = .002), but not in the change rates of fluency (variance of slope = 35.217, *p* = .426).

As is clear, learners’ speaking accuracy improved significantly, but fluency did not improve significantly. Both accuracy and fluency developed in non-linear trajectories. There existed significant individual differences in the initial level of fluency, but not in the initial level of accuracy or change rates of accuracy and fluency.

### Development of Self-Efficacy Through Online Scaffolding

The longitudinal data of self-efficacy for accuracy (SEA) and self-efficacy for fluency (SEF) were used to build linear, non-linear and free time scores LGCM. The model fit information is presented in Table 6 (non-linear LGCM of SEF was warned “PSI is not positive definite”). For SEA, the non-linear model fit best, and for SEF, free time scores LGCM fit best. Therefore, the model results of non-linear LGCM of SEA and free time scores LGCM of SEF are reported.

**Table 6:** Model fit information of LGCM of SEA and SEF.^d^

Model	AIC	BIC	SABIC	X^2^ (df)	*p*	CFI	TLI
Linear LGCM of SEA	800.193	820.066	785.586	77.669 (16)	< 0.001	0.721	0.739
Non-linear LGCM of SEA	758.941	786.041	739.022	20.370 (12)	0.060	0.962	0.953
Free time scores LGCM of SEA	774.996	802.096	755.078	38.959 (12)	< 0.001	0.878	0.848
Linear LGCM of SEF	853.521	873.394	838.914	48.892 (16)	< 0.001	0.802	0.814
Free time scores LGCM of SEF	829.451	856.551	809.533	15.921 (12)	0.195	0.976	0.970

^d^ SEA = self-efficacy for accuracy; SEF = self-efficacy for fluency.

**Fig. 3:**
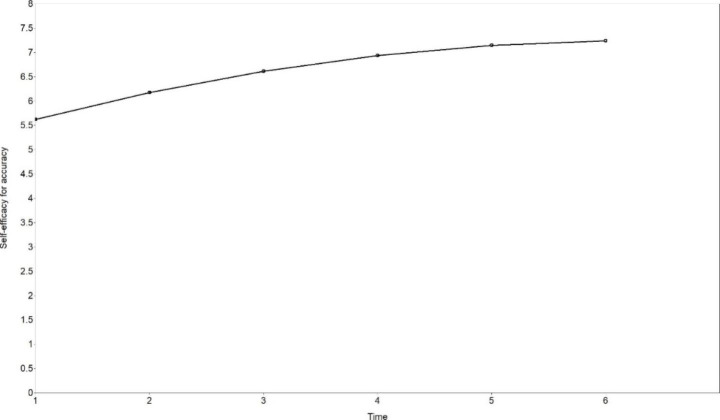
The estimated trajectory of self-efficacy for accuracy (non-linear LGCM)

The mean initial level of SEA was 5.620, a medium level (the scale was rated 1–11). The mean of the linear slope was positive (0.610, *p* < .001), and the mean of the second order slope was negative (-0.057, *p* = .024), showing that the development of SEA followed an ascending non-linear curve, and the growth rates decreased with time. It means that the greatest change of SEA took place at Time 2, but unlike the fluctuation of accuracy after Time 2, the growth of SEA was continual, just with a decreasing trend of growth rates. The estimated trajectory is shown in Fig. 3. There existed significant inter-individual differences in the initial level (mean of variance = 1.085, *p* = .030), the linear slope (mean of variance = 0.787, *p* < .001), and also the second order slope (mean of variance = 0.019, *p* < .001) of SEA.

**Fig. 4:**
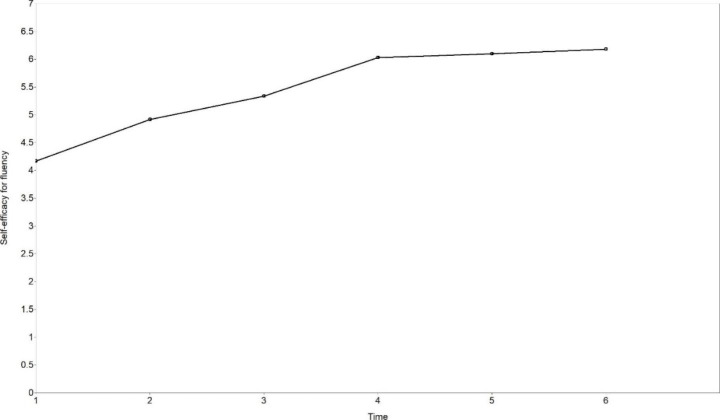
The estimated trajectory of self-efficacy for fluency (free time scores LGCM)

The mean initial level of SEF was 4.165, a lower level than SEA. The increase of SEF was significant (mean of slope = 0.750, *p* < .001). Time scores were 0.000, 1.000, 1.559, 2.482, 2.575 and 2.684, indicating non-linear development of SEF. Variations between observations were calculated based on means of slope and time scores (Table 5). The major increase of SEF happened from Time 2 to Time 4. The estimated trajectory is shown in Fig. 4. Significant inter-individual differences were found in the initial level of SEF (variance of intercept = 1.315, *p* = .002), but not in the change rates of SEF (variance of slope = 0.240, *p* = .113).

To sum up, SEA and SEF both improved significantly and followed non-linear trajectories. The greatest growth of SEA and SEF both took place at Time 2. Significant inter-individual differences were found in the initial levels of SEA and SEF, and in the change rates of SEA, but not in the change rates of SEF.

### The Dynamic Relation Between L2 Speaking Performance and Self-Efficacy

Free time scores LGCM of accuracy and non-linear LGCM of SEA were put in one LGCM with parallel processes. The model was an acceptable fit [AIC = 662.598, BIC = 727.638, SABIC = 614.793, X^2^ (df) = 72.334 (54), *p* = .049, CFI = 0.937, TLI = 0.923]. Covariance analysis shows that the linear slope of SEA was positively related with the intercept of accuracy (Cov = 0.061, *p* = .035), and the second order slope of SEA was negatively related with the intercept of accuracy (Cov = − 0.010, *p* = .027). This means that the initial level of accuracy was related to the development of SEA. A higher initial level of accuracy was related to a greater increase of SEA and a greater decrease of growth rates with time.

Free time scores LGCM of fluency and SEF were put in one LGCM with parallel processes. The model was warned “PSI is not positive definite”, so the model estimates are not reported here. Then SEF at Time 1 was put into free time scores LGCM of fluency as a covariate to find out whether the initial level of SEF influenced the initial level and change rates of fluency. The model fit well [AIC = 2358.797, BIC = 2389.510, SABIC = 2336.222, X^2^ (df) = 20.840 (16), *p* = .185, CFI = 0.983, TLI = 0.978], but no significant effect was found. In brief, the initial level of accuracy was found to relate with the development of SEA, however, the relationship between fluency and SEF was not confirmed.

## Discussion

### Development of Accuracy and Fluency Through Online Scaffolding

The significant improvement of speaking accuracy is correspondent with most longitudinal studies based on group data analysis and some case studies (Ferrari, 2012; Tonkyn, 2012; Vercellotti, 2017; Yu & Lowie, 2020). This study expands previous findings to the context of online teaching, showing that accuracy could also improve over one semester of online scaffolding. This is inconsistent with the study of Polat and Kim (2014) on an untutored immigrant, whose accuracy did not improve over one year. This difference might be explained by the differences in the context of language development (tutored/untutored). The untutored immigrant in the study of Polat and Kim (2014) mainly used the language to communicate with people, so the accuracy of language may not be as important as the expression of meaning. In contrast, the participants in the current study learned and used English mainly in English classes, and they needed to take English exams, in which accuracy was of paramount importance. The form of online scaffolding in this study may also increase their awareness of accuracy. When students expressed their ideas via QQ voice function in online class, the voice message could be heard repeatedly by anyone in the class, receiving more feedback from teachers and peers, which may cause students to pay more attention to accuracy (the form of language).

The increase of fluency was not significant, which is different from some previous studies (e.g., Tonkyn, 2012; Vercellotti, 2017). This difference might be caused by the different language context (ESL/EFL) and the different teaching context (offline/online). Unlike the target language context, learners in the current study stayed at home in China during the observation period and had no natural environment to use English at all. Different from offline courses, online courses in the current study were short of chances for effective group talk or partner talk, so learners had fewer opportunities to use English for real communication and spontaneous talk. In most oral tasks during this online teaching semester, preparation was guaranteed, so they may write down the content before talk and lost the chance to practice fluency. However, the current study is not an experimental study, so it is not safe to claim that online courses are less effective in improving one’s speaking fluency. Besides, fluency is multi-dimensional itself (Suzuki & Kormos, 2022), but only one measurement of fluency was used in this study, so the development of other aspects of fluency was not fully captured.

The non-linear development of accuracy and fluency supports previous longitudinal case studies (Evans & Larsen-Freeman, 2020; Ferrari, 2012; Polat & Kim, 2014; Yu & Lowie, 2020) and the current study makes a further attempt in analyzing non-linearity by applying free time scores LGCM. The non-linear trajectories of accuracy and fluency proves that language performance does not necessarily grow with time, and cannot be predicted in a linear way (Larsen-Freeman, 2009; Larsen-Freeman & Cameron, 2008).

Unexpectedly, the current study found no significant inter-individual differences in the initial level of accuracy or change rates of accuracy or fluency. This finding contradicts some case studies (Ferrari, 2012; Yu & Lowie, 2020). This divergence could be caused by different research methods. The current study admits that individuals’ trajectories varied to different extents, but differences in rates of change turned out to be statistically insignificant for these students. One possible reason might be the similarity of these students’ English proficiency, for they were assigned to these two classes based on a placement test. Language proficiency was found to predict accuracy in some oral tasks (Awwad & Tavakoli, 2022), and this might explain the insignificant inter-individual differences in the initial level of accuracy. These participants then received the same online scaffolding, which unintentionally attached more importance to accuracy than fluency, causing a general progress in accuracy. A further study involving more heterogeneous samples analyzed by Growth Mixture Modeling may provide more evidence for inter-individual differences in L2 development.

### Development of Self-Efficacy Through Online Scaffolding

The significant growth of efficacy is consistent with previous studies of L2 self-efficacy development (Leeming, 2017; Mills, 2009; Shirvan et al., 2018) and made contributions to the field of L2 self-efficacy development in two aspects. Firstly, this study focused on self-efficacy for speaking accuracy and fluency, which is a specific topic unexplored before. This study used a validated scale with good reliability to track the development of SEA and SEF longitudinally, providing a possible tool for relevant studies. Secondly, this study made the first attempt to track the development of L2 speaking self-efficacy in the context of online teaching, and proved that self-efficacy for accuracy and fluency could also grow significantly through online scaffolding.

The development of SEA and SEF both followed non-linear trajectories. Different from the development of “practical” accuracy or fluency, self-efficacy for accuracy and fluency never decreased over the observation period, indicating that learners became increasingly confident in the whole process, though their practical performance may not grow synchronically with their self-efficacy beliefs. Previous longitudinal studies of L2 self-efficacy mainly focused on linearity (Fryer et al., 2022; Kyo, 2022; Shirvan et al., 2018), and the finding of this study indicates that non-linearity of L2 self-efficacy development should not be ignored.

There existed significant inter-individual differences in the change rates of SEA in our study, consistent with findings from some longitudinal studies of self-efficacy (Leeming, 2017; Shirvan et al., 2018). On the one hand, this finding supports these studies and extends the study of inter-individual differences in self-efficacy development to the development of specific dimensions. On the other hand, the significant difference in SEA change rates but nonsignificant difference in SEF change rates indicate that different dimensions of self-efficacy for English speaking may present different features and are worth exploring. One interesting phenomenon is that these learners did not show significant differences in initial levels and change rates of accuracy, but they did show significant differences in the initial levels and change rates in SEA. This inter-individual difference in SEA may partly explain why the change rates of accuracy were not related to those of SEA.

### The Dynamic Relation Between L2 Speaking Performance and Self-Efficacy

The finding that the initial level of accuracy was related to SEA development supports that one’s mastery experience is a source of self-efficacy (Bandura, 1997). It means successful experiences of language performance may raise one’s self-efficacy, and unsuccessful experiences may decrease one’s self-efficacy. This study does not confirm a “one-time” relationship between mastery experience and self-efficacy, but provides evidence for a more sustainable relationship between language performance and self-efficacy development.

Unfortunately, this study found no significant relationships between the initial level of L2 performance and the initial level of self-efficacy, or between the change rates of L2 performance and the change rates of self-efficacy. This finding disagrees with most previous cross-sectional studies (e.g., Hsieh & Schallert, 2008; Mahyuddin et al., 2006; Mills et al., 2007; Noorollahi, 2021; Truong & Wang, 2019), which supported a strong relationship between self-efficacy and language performance. Cross-sectional studies draw conclusions based on a large sample at one time, so the nonsignificant relationship between initial levels of performance and self-efficacy might be partly justified by a much smaller sample size in our longitudinal study. As Lowie and Verspoor (2019) pointed out, identifying learner factors influencing language learning based on group cross-sectional data was often successful, but did not work well when tracking individuals’ trajectories longitudinally. Another longitudinal study (Zhang et al., 2020) showed that the hypothesized relationship between self-efficacy and language performance cannot be proven. Another possible reason might be the short period of observation. More longitudinal studies based on larger samples and longer observation might be needed to provide more evidence; however, the nonsignificant relationship between L2 speaking development and self-efficacy development might be explained by non-linearity and inter-individual differences found in our study.

### An Overall Discussion Based on CDST

The current study provides evidence for non-linearity, variability, inter-individual differences and dynamic relationships in L2 speaking and self-efficacy development, correspondent with the perspective of CDST (Larsen-Freeman, 1997, 2006, 2009, 2020; Larsen-Freeman & Cameron, 2008; Lowie & Verspoor, 2019).

The non-linear development of accuracy, fluency and self-efficacy found in our study not only echoes with the non-linearity feature of complex dynamic systems (Larsen-Freeman, 1997, 2011; Larsen-Freeman & Cameron, 2008; Lowie, 2017; van Geert & Steenbeek, 2005), but also demonstrates the “variability” phenomenon in language learning (Larsen-Freeman, 2009, 2011; Larsen-Freeman & Cameron, 2008; Verspoor & de Bot, 2022; Verspoor et al., 2021). Intra-individual variation, also called variability, is normal in development, and increased variability may be a precursor for a system change (Chang & Zhang, 2020, 2021; Ortega, 2011; Thelen & Smith, 1996; Verspoor & de Bot, 2022; Verspoor et al., 2021). In the current study, accuracy experienced a great growth at Time 2, but after that, the system was relatively stable with a lower level of variability, indicating that the improvement mainly took place in the early stage. This is evidence for “strong fluctuations early on” and “a smaller bandwidth of fluctuations at later moments when the systems approach stability” (Verspoor et al., 2021, p. 3). Major increases of self-efficacy for accuracy happened from Time 2, and then the growth rates gradually became lower with the passage of time. The growth of self-efficacy for fluency mainly took place from Time 2 to Time 4, and the level did not fluctuate much at Time 5 and Tome 6. Therefore, the early stage witnessed greater variability of self-efficacy, and the later stage was a relatively stable state with lower variability. This, again, provides evidence for the “variability” phenomenon in system development and indicates a system change during the observation period.

Significant inter-individual differences were found in the change rates of self-efficacy for accuracy. This finding supports the CDST perspective that individuals may have different, preferred paths and inter-individual differences should not be neglected (Chang & Zhang, 2021; Larsen-Freeman, 2006; Lowie & Verspoor, 2019). The CDST perspective considers inter-individual differences to be informative (Larsen-Freeman & Cameron, 2008). If a traditional statistical analysis (for instance, ANOVA) was used without considering individual trajectories, inter-individual differences would not be detected. However, inter-individual differences were found nonsignificant in the change rates of accuracy, fluency and self-efficacy for fluency. This does not mean that every individual developed in the same way. As Lowie and Verspoor (2019, p. 185) argued, “no two individuals will develop in exactly the same manner as development takes place in a nonlinear fashion”. If individual trajectories are visually compared, the development of these variables must be different across learners. This study does not deny individual differences in accuracy, fluency and self-efficacy for fluency development, but it can be concluded that the change rates of these variables did not differ significantly across learners. This indicates that for these learners receiving the same instructions, despite intra-individual variability and inter-individual differences in trajectories, some features are universal in the process of development. This universality is as informative as inter-individual differences, because it would be interesting to further investigate why learners are similar in one aspect, but different in another, which can be further studied in the future.

It is found in our study that the initial level of accuracy related to the change rates of self-efficacy for accuracy, confirming the CDST perspective that the relation between elements should be viewed dynamically (Larsen-Freeman, 2020; Larsen-Freeman & Cameron, 2008). Although in the current study, the initial level of language performance was not found to correlate with the initial level of self-efficacy, it was found to correlate with the change rates of self-efficacy. This finding supports the importance of conducting longitudinal studies of dynamic relations between L2 development and learner factors. However, the current study did not confirm the relationship between the change rates of L2 speaking performance and those of self-efficacy. This nonsignificant relationship might be explained by non-linearity and inter-individual differences in L2 speaking and self-efficacy development. As reported above, accuracy, fluency, self-efficacy for accuracy and self-efficacy for fluency in our study all developed in non-linear trajectories; and though greater variability all took place in an early stage, the non-linear trajectories of these four variables were very different. Both accuracy and self-efficacy for accuracy experienced the greatest increase at Time 2, but accuracy fluctuated with small variability after that, while self-efficacy for accuracy never decreased and kept increasing with lower growth rates. Besides, while there was no significant inter-individual difference in accuracy initial levels and change rates, the difference was significant in the initial levels and change rates of self-efficacy for accuracy. All these inconsistencies could well explain the insignificant relation between the initial levels and change rates of accuracy and self-efficacy for accuracy. For fluency and self-efficacy for fluency, a greater inconsistency existed. Fluency did not improve significantly, while self-efficacy for fluency improved significantly, so the “practical” fluency did not grow synchronically with learners’ self-belief. In short, when non-linearity and inter-individual differences are considered, the relationship between language development and learner factors becomes more complex.

## Conclusions

By tracking the development of 45 Chinese learners’ English speech and self-efficacy over one semester of online course, this longitudinal study found that speaking accuracy, self-efficacy for accuracy and self-efficacy for fluency all improved significantly, but speaking fluency did not. These four variables all developed in non-linear trajectories. There existed significant individual differences in the change rates of self-efficacy for accuracy. The initial level of accuracy was related to the development of self-efficacy for accuracy.

These findings have some pedagogical implications for online scaffolding. Firstly, results show that, through one semester of online course, students’ speaking accuracy improved significantly. This suggests that the current online scaffolding may facilitate students’ progress in accuracy by providing proper support before tasks, enabling every student to present oral production after preparation, and giving adequate and individualized feedback. Secondly, through the online scaffolding in the present study, student’ fluency did not improve significantly. This might be caused by a variety of factors, but admittedly, the forms of online scaffolding adopted in the current study failed to provide context for spontaneous talk and natural communication. Nowadays, teachers might want to use teaching software to set up sub-conference rooms to enable group discussion, and at the same time, they can get into any sub-conference room to monitor student progress and provide proper help. Some useful virtual speaking platforms, if developed properly, may also help create a speaking context. It is recommended that language teachers guarantee spontaneous talk and natural communication whenever it is possible for them to help students improve their fluency. Thirdly, students’ self-efficacy for accuracy and fluency increased through online scaffolding. This, to some extent, relieves language teachers’ worry that students’ confidence in spoken language might decline through online teaching. By designing activities that involve adequate oral production and feedback, teachers could help enhance students’ confidence in developing their speaking proficiency. Finally, the current study confirmed the non-linear development in students’ speaking ability. Since scaffolding should be provided according to students’ current and expected level, dynamic tracking of students’ ability is of great importance (Smit et al., 2017). Despite our effort of tracking students’ speaking ability and self-efficacy, we did not provide the online scaffolding based on the dynamic change of levels. From our study, it is clear that language teachers need to integrate scaffolding with dynamic assessment of students’ ability, which develops in a non-linear way.

We need to acknowledge the limitations, one of which is of a small sample size. As a result, we were not able to incorporate the dynamic self-efficacy scores as time-variant covariates into the model or further explore inter-individual differences using Growth Mixture Modeling analysis. The other limitation is that we only adopted one measure for accuracy or fluency, which may not present the whole picture. The third possible limitation is that only quantitative data were used, so the non-linear development and inter-individual differences were not able to be well explained. Researchers are recommended to combine quantitative and qualitative data in studies of speech performance and learner factors (Sun & Zhang, 2022; Zhang et al., 2022).

Despite these limitations, this study made the first attempt to analyze L2 speaking accuracy and fluency development related to self-efficacy by using LGCM, showing that LGCM could be a proper analysis technique for modeling group trajectories of speaking accuracy and fluency; Clearly, a larger sample size is highly recommended for analyzing dynamic relationships. This study also provides evidence for non-linearity, variability and inter-individual differences in the development of L2 speaking and self-efficacy through online scaffolding. The findings partly confirm the dynamic relationships between self-efficacy and L2 performance. They echo with the CDST perspective and add new evidence from the context of online teaching. Most importantly, the rarely explored issue, the dynamic relationship between L2 speaking CAF development and individual difference factors, was brought to the fore. Nonetheless, further work is needed along this line.
